# Quantification of Vitamin A in Edible Oils: Comparison of Portable Device iCheck Chroma3 to High-Performance Liquid Chromatography

**DOI:** 10.1007/s12161-024-02613-w

**Published:** 2024-03-23

**Authors:** Susana A. Palma-Duran, David Morgan, Emilie Combet

**Affiliations:** 1 School of Medicine, College of Medical, Veterinary and Life Sciences, The University of Glasgow, Glasgow, UK; 2 Department of Food Science, Research Center in Food and Development A.C., Hermosillo, Mexico; 3 Global Alliance for Improved Nutrition (GAIN), Geneva, CH Switzerland

**Keywords:** Vitamin A, Spiked and fortified oils, Portable device, Repeatability, Linearity, Limits of agreement, HPLC, iCheck Chroma3

## Abstract

Fortification of edible oil with vitamin A is a widely adopted intervention to minimize the effects of vitamin A deficiency in vulnerable groups and mitigate some of its deleterious consequences. Regulatory monitoring is an important prerequisite to ensure that the fortification program is implemented effectively. Standard laboratory analysis methods for vitamin A in oils to assess adequate addition levels remain expensive and time-consuming. Portable testing devices are relatively less expensive in terms of capital investment and cost per test. However, the reliability of results needs to be assured to ensure acceptability and confidence. This study compared a portable device to high-performance liquid chromatography (HPLC) in terms of quantification of vitamin A in both spiked and commercially fortified oils. Nine oils (soybean, palm, cottonseed, rapeseed, corn, peanut, coconut, sunflower, and rice bran oils) were selected and spiked with retinyl palmitate at six different concentrations, and 112 commercially fortified oils were quantified for their vitamin A content using both methods. A good indicator of intra-day and inter-day repeatability (< 10% CV) was obtained for the measurement of vitamin A in the spiked oils for both methods, which denotes a high agreement between them. Vitamin A recoveries were 97–132% for HPLC and 74–127% for the portable device. A strong positive correlation, *r* = 0.88, is observed between the two methods for the quantification of vitamin A in the commercially fortified oils. The portable device provides a relatively low-cost, quick, and user-friendly alternative to HPLC.

## Introduction

Vitamin A deficiency (VAD) is an issue of global public health concern, affecting about three billion people, mainly in developing countries, with a third of all those affected being children between 6 months and 5 years old (https://data.unicef.org/topic/nutrition/vitamin-a-deficiency/#), (Renaud et al. 2013; Akhtar et al. 2013; Zhao et al. 2022). Vitamin A—a fat-soluble vitamin—plays a vital role in health functions such as improved cell division, immunity cell growth, differentiation of cells, development of embryos, and general growth (McLaren and Frigg 2001). Vitamin A deficiency can increase susceptibility to infections, visual impairments, and eventually blindness. Severe VAD in adults (especially pregnant women) and young children is a major contributing factor to mortality in these vulnerable groups (West 2004). Food fortification remains one of the most effective policies for improving vitamin A intake, thus preventing VAD, and some foods like flour, sugars, and oils have been identified as the most appropriate vehicles for food fortification programs (West and Darnton-Hill 2008). Plant-based oils (particularly palm, soya, and sunflower seed oils) are commonly consumed in many African and Asian countries, which are also affected by prevalent VAD (Walters et al. 2019; Organization WH 2009). Oils are a cost-effective matrix for vitamin A fortification and enable homogeneous vitamin A dispersion. The increased stability during fortification also delays the oxidation of the vitamin (Renaud et al. 2013; Dary and Mora 2002; Rohner et al. 2011).

A significant factor in a national food fortification program’s success is the ability to detect whether the fortified food has an adequate micronutrient level as specified in the relevant national standard. Data about the quality, coverage, and impact of fortification programs are essential. This data is needed for regulatory monitoring and the concomitant or resultant programmatic course corrections. Laboratory analyses have been developed and are frequently deployed to quantify micronutrients in fortified foods. Current gold standard methods of analyzing vitamin A are methodologically complex and expensive, making it difficult to ensure a consistent combination of machinery, reagents, and human resource capacity (Oliver and Palou 2000; Zhang 2018; Wayenbergh et al. 2023; Rimkus et al. 2022).

To enhance rapid analyses and regulatory monitoring of vitamin A fortified oils in the field, simple portable devices that are robust, field- and user-friendly, and can be used by technicians with basic training are potentially useful. The iCheck Chroma3 is a product developed by BioAnalyt GmbH (Teltow, Germany). Its predecessor, the iCheck Chroma, was applicable to a limited number of oils such as palm, sunflower, corn, peanut, rapeseed, and coconut (Rohner et al. 2011) and did not provide accurate results for other important oil types such as soybean and cottonseed (Renaud et al. 2013). The iCheck Chroma3 sought to improve analytical performance with soybean and cottonseed oils.

The objective of this study was to validate the portable device iCheck Chroma3 (BioAnalyt GmbH) against an HPLC reference method (carried out by an independent laboratory) using oils spiked with retinyl palmitate and market samples of commercially fortified oils obtained in Nigeria.

## Materials and Methods

### Materials and Reagents

Analytical grade retinyl palmitate, soybean, palm, cottonseed, rapeseed, corn, peanut, and coconut oil were purchased from Sigma-Aldrich (Poole, Dorset, UK). Sunflower oil (analytical grade) was purchased from Insight Biotechnology Limited (Wembley, UK). Commercial edible-grade rice bran oil was purchased from British Essentials (Alfa one brand, UK). The iCheck Chroma3, a portable device to quantify vitamin A, was provided by BioAnalyt (Teltow, Germany).

### Oil Fortification

A stock solution was prepared by mixing 0.9004 g of retinyl palmitate with 164.1 g of rapeseed oil overnight at 6 rpm in a dark bottle to achieve a final concentration of 3009.6 mg RE/kg. The stock solution was used for oil fortification to reach concentrations of 0, 3.0, 5.0, 10, 15, 20, and 30 mg RE/kg—with oils and spiked solutions carefully weighed on a precision balance (Fisher Scientific, analytical balance). Spiked oils were mixed at 100 rpm for 3 h, as recommended on the label. The spiked oils were stored at room temperature, ~ 18 °C (rapeseed and palm oils at 4 °C), in the dark prior to analysis. Coconut and palm oils were gently heated (50 °C, 20 min) to homogenize the content before fortification and analysis. Oils were analyzed within 2 weeks of fortification. The final concentrations of the spiked oil samples are shown in Table 1. Throughout this paper, concentrations are reported as retinol equivalent (RE) in mg per kg of oil.

**Table 1:** Concentration of the selected oil samples (mg RE/kg) fortified with retinyl palmitate

Nominal concentration in mg RE/kg	Soybean	Palm	Cottonseed	Sunflower	Rapeseed	Corn	Peanut	Rice bran	Coconut
0	0.0	0.0	0.0	0.0	0.0	0.0	0.0	0.0	0.0
3	3.9	3.2	3.3	3.0	3.0	2.9	3.0	2.8	3.1
5	4.9	5.3	5.2	5.0	5.0	5.3	5.4	5.3	4.9
10	10.6	10.1	11.0	10.0	9.7	9.8	9.6	9.7	10.9
15	15.9	14.1	15.1	14.8	15.3	15.1	16.0	14.6	15.1
20	19.3	20.7	20.3	20.7	21.7	20.5	20.1	19.9	20.1
30	30.4	29.2	31.1	29.3	29.8	31.3	30.9	30.1	30.6

### Commercially Fortified Oils

A total of 112 selected oils which had been commercially fortified with vitamin A were quantified for their vitamin A contents. These commercially fortified oil samples were collected in Nigeria from markets and households May–June 2017 by the Global Alliance for Improved Nutrition (GAIN). These samples of fortified oils were sent to BioAnalyt in July 2017 and then to the Human Nutrition laboratory (University of Glasgow) in August 2017. The test oils were delivered in plastic bottles, stored at room temperature (~ 20 °C) in the dark before analysis. Any solid test oil was gently heated (50 °C, 20 min) to homogenize the content before analysis. The vitamin A concentration in the test oils was measured using the two quantification methods: iCheck Chroma3 at the University of Glasgow and HPLC at Intertek, Germany.

### Quantification of Vitamin A in Spiked Oils by High-Performance Liquid Chromatography (HPLC)

Portions of all oil samples (~ 20 mL) were shipped to a commercial, independent, laboratory (Intertek, Germany). These oils were analyzed by HPLC, with method replication carried out every ten samples on a different sequence (these replicates were used to calculate the inter-assay precision, “Procedure for Assessment of the Methods”). Briefly, oil samples (1 g) were filled up to 10 mL with methyl tert-butyl ether in dibuthylhydroxytoluene (1 g/L). After thoroughly shaking the solution, methanol was added (1:1) and injected (20 µL) to the HPLC. Vitamin A was analyzed on a Thermo Scientific Accela 600 HPLC (Waltham, USA), coupled to a fluorescence detector (*λ*_ex_, 325 nm; *λ*_em_, 480 nm, Thermo Scientific Surveyor FL Plus, Serial number 650212). The vitamin A was separated using a Thermo hypersil GOLD C_18_ (150 × 4.6 mm, 3 µm). The mobile phase included methanol (solvent A) and methyl tert-butyl ether (solvent B), programmed as follows: 0–10.0 min: 0% B, 10–14 min: 100% B, 14–19 min: 0% B, at a flow rate of 1.0 mL/min. Retinyl palmitate in methanol was used as an external calibrator. The limit of quantification was 0.55 mg RE/kg as defined by the commercial independent laboratory.

### Quantification of Vitamin A in Spiked Oils by the Portable Device—iCheck Chroma3

The vitamin A concentrations of oil samples were quantified by a single user using the device following the instructions in iCheck Chroma3 User Manual.

The iCheck Chroma3 is a portable device with dimensions 11 × 4 × 20 cm (*W* × *H* × *L*) and weighing 450 g. The device has a concentration range of 3.00–30.00 mg retinol equivalent (RE)/kg (or 10.00–100.00 international units (IU)/g). This range was previously and independently defined by the manufacturer based on linearity of recovery of added vitamin A greater than *R*^2^ = 0.95 and a deviation from expected vitamin A under ± 20%. This means that samples above 30.00 mg RE/kg will have to be diluted in refined unfortified oil. The iCheck Chroma3 is powered by NiMH rechargeable batteries (AA 1.2 or 1.5 V) to facilitate use in the field. The time per analysis is typically less than 2 min. The device works best at room temperature (20–30 °C) with no direct exposure to sunlight (https://www.bioanalyt.com/product/icheck-chroma/).

Briefly, oil samples (100 µL) were injected manually into the supplied reagent vial prefilled with 2 mL of antimony trichloride in chloroform. The vial was rapidly inverted twice and immediately inserted in the iCheck Chroma3 device for measurement by the user. Vitamin A concentration determined by iCheck Chroma3 is based on the timed reaction of antimony trichloride in chloroform with the double bond of retinol (Carr-Price reaction) that forms anhydroretinylic and retinylic cations producing a blue color, proportional to vitamin A. The device measures the progression of the color development at different wavelength, one specific for the blue color and others to account for background color that may interfere with blue color reading. The concentration of vitamin A is reported in mg RE/kg. All measurements were performed at room temperature (20 °C), stored in the device, and transferred to a personal computer as a.txt file at the end of each session.

### Procedure for Assessment of the Methods

Recovery, accuracy, and precision were evaluated as part of method validation in the iCheck Chroma3 and the reference method (HPLC). Spiked oil samples and blanks were included in the parameter validation. Recovery and accuracy were assessed at 3.0, 5.0, 10.0, 15.0, 20.0, and 30.0 mg RE/kg in all nine selected oils.

Recovery (Eq. 1) was calculated by dividing the value obtained by the analytical methods (*A*) to the nominal concentration of the spiked oil sample (*B*) and presented in percentage.

1 $$\mathrm{Recovery\;}(\mathrm{\%})= \frac{A}{B} \times 100$$

The accuracy (Eq. 2) of the measurements for the two methods of analysis was determined using the root mean square error (RMSE) calculated as

2 $$\mathrm{RMSE }=\sqrt{\frac{\sum {\left(Y{\text{exp}}-Y\right)}^{2}}{n}}$$

where *Y*_exp_ are the expected vitamin A values, *Y* are the measured values for each method of analysis, and *n* is the number of analysis.

The concentrations of vitamin A in the spiked oils were related to the concentration of retinyl palmitate [RP] in terms of the response *R* obtained for both HPLC and the iCheck Chroma3 portable device. The relation can be expressed as a linear equation in the form

3 $$R = {a}_{0} \times [RP] + {b}_{0}$$

where *a* is the gradient or sensitivity and *b* is the intercept on the *y*-axis (https://data.unicef.org/topic/nutrition/vitamin-a-deficiency/#).

Precision was divided into intra-day and inter-day repeatability. The intra-day repeatability was determined by measuring five selected oils at a concentration of 15 mg RE/kg in duplicate on the same day, for both reference HPLC method and iCheck Chroma3. The inter-day repeatability of the iCheck Chroma3 was determined by measuring four selected oils at a concentration of 15 mg RE/kg on three different days. For the reference method, the inter-day repeatability was determined by measuring four selected oils at 10 mg RE/kg concentrations on different occasions.

Bland–Altman plots were generated to assess the agreement between the two methods by plotting the differences between the methods (*y-axis*) against the mean of the methods (*x-axis*) (Altman and Bland 1983). Limits of agreement were calculated as the differences in methods ± 2 SD of the differences in paired measurements. Linear regression analysis was used to estimate the association between the analysis methods of vitamin A. The linear regression analysis was performed by estimating the association between the differences in the methods (dependent variable) and the mean of the methods (independent variable). The association between the two methods was also examined by Pearson’s correlation coefficients.

Data processing and statistical analysis were performed using Excel 2013 (Microsoft, USA, 2017).

## Results

### Linearity and Accuracy

A detailed description of the linearity and accuracy between the oils is found in Table 2.

**Table 2:** Linearity and accuracy of spiked oils using HPLC and iCheck Chroma3

Oils	[RP]	HPLC			iCheck Chroma3		
		Linearity	*R*^2^	Accuracy	Linearity	*R*^2^	Accuracy
Soybean	3–30	*R*_HPLC_ = 1.0525 × [RP] + 0.1293	0.999	1.04	*R*_iCheck_ = 0.7783 × [RP] + 0.0685	0.9973	3.70
Palm	3–30	*R*_HPLC_ = 0.9782 × [RP] + 0.2408	0.9989	0.34	*R*_iCheck_ = 0.9875 × [RP] − 0.8879	0.9905	1.36
Cottonseed	3–30	*R*_HPLC_ = 1.1027 × [RP] − 0.2134	0.9992	1.76	*R*_iCheck_ = 0.9233 × [RP] − 0.7146	0.9959	2.16
Sunflower	3–30	*R*_HPLC_ = 1.0967 × [RP] + 0.1806	0.9991	1.39	*R*_iCheck_ = 1.1176 × [RP] + 0.4200	0.9995	1.85
Rapeseed	3–30	*R*_HPLC_ = 1.0986 × [RP] + 0.3890	0.9992	2.20	*R*_iCheck_ = 0.9393 × [RP] + 0.5291	0.9799	1.37
Corn	3–30	*R*_HPLC_ = 0.972 × [RP] + 1.5338	0.9981	1.17	*R*_iCheck_ = 1.0007 × [RP] − 1.4907	0.997	1.56
Peanut	3–30	*R*_HPLC_ = 1.0149 × [RP] − 0.2353	0.9998	0.10	*R*_iCheck_ = 1.0354 × [RP] + 0.8359	0.9983	1.33
Rice bran	3–30	*R*_HPLC_ = 1.0613 × [RP] + 0.8706	0.9976	1.67	*R*_iCheck_ = 0.8954 × [RP] + 1.8695	0.9987	0.83
Coconut	3–30	*R*_HPLC_ = 0.9997 × [RP] + 0.1466	0.9988	0.25	*R*_iCheck_ = 1.0028 × [RP] + 0.5879	0.9955	0.74

*RP *retinyl palmitate is expressed in mg RE/kg

Both methods have excellent linearity at *R*^2^ above 0.98. Previous studies (https://data.unicef.org/topic/nutrition/vitamin-a-deficiency/#) asserted that when comparing two analytical methods using the RMSE (Eq. 2), the method with the lower values has higher accuracy. This suggests that (as expected) the HPLC was relatively more accurate than the iCheck Chroma3 in the vitamin A measurements of the oils.

### Comparison of the Recoveries of the Spiked Oils (HPLC vs iCheck Chroma3)

Reference HPLC methods used to determine the vitamin A recovery in the nine selected oils ranged between 96.8% for peanut oil (at a concentration 5 mg RE/kg) and 132.1% (at a concentration 5 mg RE/kg) for rice bran oil (except for corn oil with 166.9% at 5 mg RE/kg) as shown in Table 3 (A).

**Table 3:** Vitamin A recovery (%) of the spiked oils using (A) HPLC reference method and (B) iCheck Chroma3 portable device

Concentration in mg RE/kg^a^	Soybean		Palm	Cottonseed	Sunflower	Rapeseed	Corn		Peanut		Rice bran	Coconut
(A)												
Blank	–		–	–	–	–	–		–		–	–
3.0	100.5		103.7	108.9	112.5	110.7	166.9		101.6		132.1	101.9
5.0	105.5		99.6	108.8	113.2	114.2	117.0		96.8		122.2	101.0
10	107.8		99.1	109.3	111.0	112.2	113.8		99.1		115.5	105.3
15	116.1		100.3	108.8	113.6	115.9	109.6		103.0		111.8	100.8
20	106.1		101.2	106.7	109.4	111.5	107.0		100.1		110.6	100.6
30	104.7		97.5	110.5	109.5	110.6	100.8		98.4		103.9	102.7
(B)												
Blank	–	–		–	–	–		–		–	–	–
3.0	85.4	nd*		nd*	126.0	nd*		nd*		nd*	nd*	106.9
5.0	86	84.3		93.0	118.6	75.9		79.4		122.1	127.2	115.7
10	73.7	85.2		79.5	115.2	102.3		77.2		111.2	108.2	111.2
15	75	89.7		100.5	116.7	110.0		89.8		106.6	100.5	100.2
20	79.8	101.8		87.5	113.0	97.3		96.0		109.1	99.9	103.0
30	78.7	93.3		93.8	nd**	92.4		94.6		nd**	nd**	nd**

^a^The levels of fortification (mg RE/kg) of the oils are approximates; the actual concentrations are shown in Table 1

*Recovery was not calculated because the concentration of the oil was below 3 mg RE/kg

**Recovery was not calculated because the concentration of the oil was above 30 mg RE/kg

The vitamin A recoveries (%) for the spiked oils using the iCheck Chroma3 device were relatively lower than the reference HPLC methods. These recoveries, shown in Table 3 (B), ranged between 73.7 and 127.2%, respectively, for soybean oil (at concentration 15 mg RE/kg) and rice bran oil (at concentration 5 mg RE/kg).

The iCheck Chroma3 device has a measurement range from 3 to 30 mg RE/kg; hence, it cannot accurately quantify concentrations lower than 3 mg RE/kg and higher than 30 mg RE/kg.

### Intra-Day and Inter-Day Repeatability

#### HPLC Method

The intra-day and inter-day repeatability of the oils was calculated as coefficients of variations. The coefficient of variations (CV) of the five selected oils spiked at 15 mg RE/kg was 1.53% for the reference HPLC method.

The inter-day repeatability of the HPLC method determined for the four selected oils spiked at 10 mg RE/kg showed a low variation with 4.41% CV.

#### iCheck Chroma3

The coefficient of variation of the five selected oils spiked at 15 mg RE/kg for repeatability was low with 3.85%.

The inter-day repeatability of the portable device determined between 3 days of the four selected oil spiked at 15 mg RE/kg showed a low variation with a CV of 3.48%. According to the manufacturer, a maximum CV observed during the internal assessment of the inter-day repeatability of the method was 13.5% (Zhang 2018).

### Comparison Between HPLC and iCheck Chroma3—Spiked Oil Samples

**Fig. 1:**
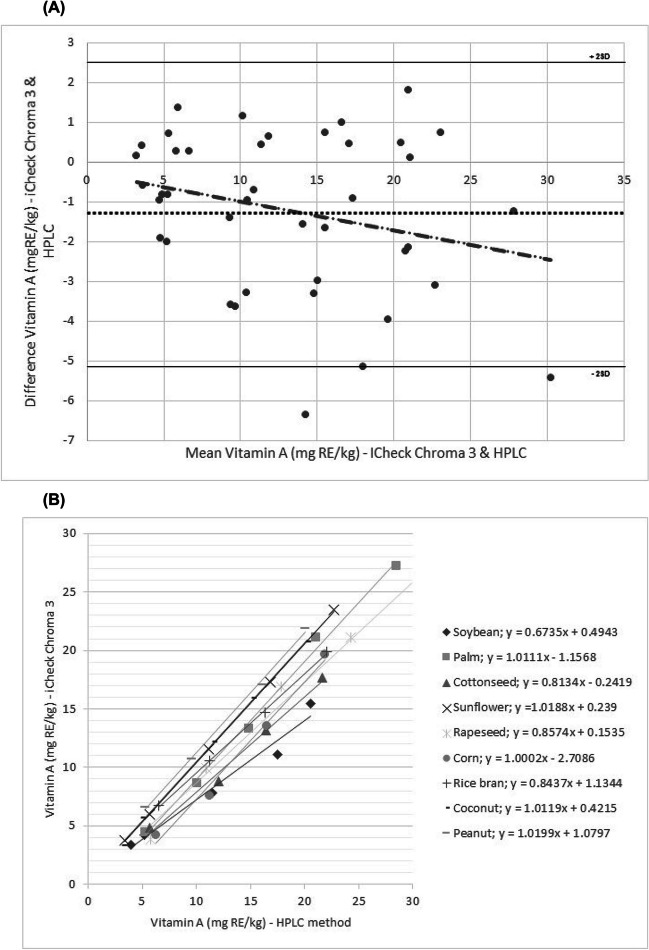
Comparison of the iCheck Chroma3 portable device and HPLC reference method. **A** Quantification of vitamin A in the nine selected spiked oils at six concentrations (3, 5, 10, 15, 20, and 30 mg RE/kg) using Bland and Altman plots at 95% limits of agreement (± 2 SD). **B** Linearity for vitamin A quantification in the nine selected spiked oils at six concentrations from 3 to 30 mg RE/kg between the iCheck Chroma3 and HPLC (*r* = 0.93, *p* < 0.001)

The degree of agreement between vitamin A quantification in the selected spiked oils by the reference method and the iCheck Chroma3 device is shown in Fig. 1. The mean difference between the methods (bias) was low with a low underestimation of 1.22 mg RE/kg by the iCheck Chroma3 portable device compared to the HPLC method. The limits of agreement had an upper limit of 2.69 mg RE/kg and a lower limit of − 5.12 mg RE/kg. There was a strong correlation between the portable device and the HPLC method for vitamin A quantification. A good linearity was observed between the methods for all the spiked oils (Fig. 1B.).

### Comparison Between HPLC and iCheck Chroma3—Commercially Fortified Oils

The levels of vitamin A for the commercially fortified oils were determined using HPLC and iCheck Chroma3 portable device. The oils had a median vitamin A concentration of 7.02 mg RE/kg with an interquartile range (IQR) of 6.75 mg RE/kg using the iCheck Chroma3 portable device. Comparably lower vitamin A concentrations were obtained using the HPLC reference method (5.70 mg RE/kg, IQR 6.30 mg RE/kg).

**Fig. 2:**
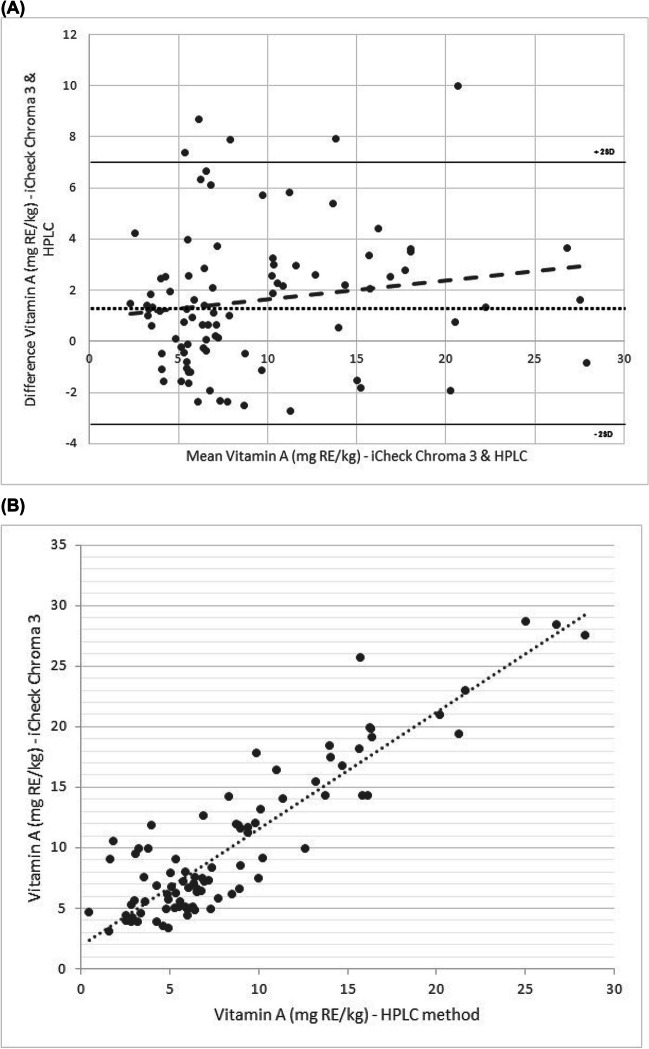
Comparison of the iCheck Chroma3 portable device and HPLC reference method for test oils (commercially fortified oils). **A** Quantification of vitamin A for the fortified commercial oils (*n* = 89), using Bland–Altman plots. The black round dotted line represents the mean difference between the methods (bias). The solid lines represent the limits of agreement (± 2SD standard deviations). The dashed line represents the linear regression analysis. **B** Correlation of vitamin A quantification in the commercially fortified oils for HPLC and iCheck Chroma3, *R*.^2^ = 0.81, and Pearson’s correlation (*r*) of 0.90 (*p* < 0.001)

The degree of agreement between vitamin A quantification in the commercially fortified oils by the reference method and the portable device is shown in Fig. 2. The mean difference between the methods was low, with an overestimation of 1.60 mg RE/kg by the portable device compared to the HPLC method. The upper limit of agreement was 6.97 mg RE/kg, and the lower limit was − 3.76 mg RE/kg. The linear regression analysis showed no apparent bias (95% CI =  − 0.06 to 0.04, *p* = 0.62, *R*^2^ = 0.0025) between the two methods for vitamin A quantification.

The equation for the correlation was *y* = 0.9639*x* + 1.9081 with a corresponding *R*^2^ of 0.81. The Bland–Altman plot (Fig. 2A) shows some points outside the + 2 SD line. No such outliers were excluded in the analyses for this study.

## Discussion

Food is fortified to provide cost-effective nutritious food to improve the micronutrient status of vulnerable groups. It is thus vital to have laboratory methods to quantify the level of micronutrient fortification reliably (Mutuku et al. 2020). Standard methods are limiting in terms of financial investment for the equipment and require, at the same time, highly trained technicians to run the time-consuming methods (Mutuku et al. 2020). This warrants using and validating rapid, inexpensive, and easy-to-use portable solutions like the iCheck Chroma3 portable device.

The intra-day and inter-day repeatability for the spiked oils using both methods of analyses expressed as % coefficients of variation was below 15%, with the HPLC methods having rather low coefficients of variation (“Intra-Day and Inter-Day Repeatability”). This corresponds to a relatively high agreement between the two methods of analysis. This study reported a recovery of 96.8–132.1% for HPLC and 73.7–127.2% for iCheck Chroma3 portable device. These recovery rates were well within those obtained from a previous study comparing HPLC and the iCheck Chroma3 methods (Rohner et al. 2011).

The quantification of vitamin A in the commercially fortified oils using the HPLC reference method and iCheck Chroma3 portable device showed a high degree of agreement, with a Pearson correlation (*r*) value of 0.90. This relates to a strong positive correlation between the two methods of analyses for vitamin A, although the iCheck Chroma3 portable device had relatively higher values for the quantification of vitamin A in the commercially fortified oils.

In validating an analytical method, careful consideration has to be given to the detection and quantification limits. These are expressed as the lowest concentration of a compound detected by the method (LOD) and the lowest concentration of the compound to enable its quantification (LOQ). The linear range of vitamin A for the iCheck Chroma3 portable device is 3.0–30.0 mg RE/kg for most oils, whereas HPLC methods can detect concentrations lower than 3.0 mg RE/kg and higher than 30.0 mg RE/kg. Although concentrations lower than 3.0 mg RE/kg cannot be measured with the iCheck Chroma3 device, concentrations higher than 30.0 mg RE/kg can be measured. This is achieved by diluting the oil with unfortified refined oil of the same kind and then multiplying the results by the dilution factor to obtain the true value of vitamin A in the oil sample (Renaud et al. 2013; Oliver and Palou 2000). The linear range of the iCheck Chroma3 device is, however, not a limiting factor for its use in fortification programs for oils. The reason is that recent guidelines for vitamin A fortification in oils published by WHO/FAO put the fortification levels at 5.0–15.0 mg/kg (Wayenbergh et al. 2023), which is within the device’s detection range.

## Conclusion

The portable iCheck Chroma3 device quantifies vitamin A in oils (3–30 mg RE/kg) with comparable results to the HPLC reference method. The two methods showed comparable intra-day and inter-day repeatability and linearity. There is relatively low bias and small limits of agreement between methods, which identifies the portable device as a reliable instrument for quantifying vitamin A in oil samples from a variety of plant sources. The iCheck Chroma3 portable device can be used as a fast, reliable, and cost-effective method for analyses of vitamin A in oils. Further studies can be done into its validation for other oils not within its current scope of analysis. All oils that are commercially produced and considered fortifiable were tested here, and there is scope for further development to minimize variability due to user-generated error (e.g., related to mixing and timing of the reaction reading).

## Data Availability

The data that support the findings of this study are available from the corresponding author upon reasonable request.

## Declarations

### Disclaimer

The funder (Bill and Melinda Gates Foundation) played no role in the study design, data collection and analysis, decision to publish, or preparation of the manuscript.

## References

[CR1] Akhtar S, Ahmed A, Randhawa MA, Atukorala S, Arlappa N, Ismail T, Ali Z (2013). Prevalence of vitamin A deficiency in South Asia: causes, outcomes, and possible remedies. J Health Popul Nutr.

[CR2] Altman DG, Bland JM (1983). Measurement in medicine: the analysis of method comparison studies. J R Stat Soc: Ser D (The Statistician).

[CR3] Dary O, Mora JO (2002). Food fortification to reduce vitamin A deficiency: International Vitamin A Consultative Group recommendations. J Nutr.

[CR4] McLaren DS, Frigg M (2001) Sight and life guidebook on vitamin A in health and disease. Task Force Sight and Life Basel, Switzerland

[CR5] Mutuku JM, Mwaniki MW, Onjong H, Michira J (2020). The biofortification continuum: implications for food and nutrition security in developing countries. Afr J Food Agric Nutr Dev.

[CR6] Oliver J, Palou A (2000). Chromatographic determination of carotenoids in foods. J Chromatogr A.

[CR7] Organization WH (2009) Global prevalence of vitamin A deficiency in populations at risk 1995–2005: WHO global database on vitamin A deficiency

[CR8] Renaud C, Berger J, Laillou A, Avallone S (2013). Quantification of vitamin A in fortified rapeseed, groundnut and soya oils using a simple portable device: comparison to high performance liquid chromatography. Int J Vitam Nutr Res.

[CR9] Rimkus GG, Schubert M, Morgan D, Jungjohann S (2022). Rapid direct analysis of retinyl palmitate (vitamin A) in fortified vegetable oils by HPLC-FLD. Food Additives & Contaminants: Part A.

[CR10] Rohner F, Frey SK, Mothes R, Hurtienne A, Hartong S, Bosso PE, Bui M, Schweigert FJ, Northrop-Clewes C (2011). Quantification of vitamin A in palm oil using a fast and simple portable device: method validation and comparison to high-performance liquid chromatography. Int J Vitam Nutr Res.

[CR11] Van Wayenbergh E, Verheijen J, Langenaeken NA, Foubert I, Courtin CM (2023). A simple method for analysis of vitamin A palmitate in fortified cereal products using direct solvent extraction followed by reversed-phase HPLC with UV detection. Food Chem.

[CR12] Walters D, Ndau E, Saleh N, Mosha T, Horton S (2019). Cost-effectiveness of sunflower oil fortification with vitamin A in Tanzania by scale. Matern Child Nutr.

[CR13] West K (2004). Vitamin A deficiency as a preventable cause of maternal mortality in undernourished societies: plausibility and next steps. Int J Gynecol Obstet.

[CR14] West KP, Darnton-Hill I (2008) Vitamin A deficiency. Nutr Health Dev Countries 377–433

[CR15] Zhang Y, Zhou W-E, Yan J-Q, Liu M, Zhou Y, Shen X, Ma Y-L, Feng X-S, Yang J, Li G-H (2018). A review of the extraction and determination methods of thirteen essential vitamins to the human body: an update from 2010. Molecules.

[CR16] Zhao T, Liu S, Zhang R, Zhao Z, Yu H, Pu L, Wang L, Han L (2022). Global burden of vitamin A deficiency in 204 countries and territories from 1990–2019. Nutrients.

